# 2-(2-Chloro-3-quinol­yl)-3-phenyl­thia­zolidin-4-one

**DOI:** 10.1107/S1600536809041543

**Published:** 2009-10-17

**Authors:** Wei-Wei Liu, Ji-You Sun, Li-Juan Tang, Yue-Qiang Zhao, Hong-Wen Hu

**Affiliations:** a School of Chemical Engineering, Huaihai Institute of Technology, Lianyun-gang Jiangsu 222005, People’s Republic of China; b Department of Chemistry, Nanjing University, Nanjing Jiangsu 210093, People’s Republic of China

## Abstract

In the title compound, C_18_H_13_ClN_2_OS, the thia­zolidinone ring is slightly distorted and adopts a envelope conformation. The basal plane is nearly perpendicular to the quinoline ring, forming a dihedral angle of 86.1 (1)°, and makes a dihedral angle of 14.9 (1)° to the benzene ring. The benzene ring is also nearly perpendicular to the quinoline ring, forming a dihedral angle of 89.4 (1)°. In the crystal, non-classical C—H⋯O and C—H⋯N hydrogen bonds link the mol­ecules, forming polymers along *b*.

## Related literature

For the biological activity of thia­zolidinone derivatives, see: Abd Elhafez *et al.* (2003 ▶); Kuecuekguezel *et al.* (2006 ▶); Shih & Ke (2004 ▶); Subudhi *et al.* (2007 ▶); Srivastava *et al.* (2006 ▶).
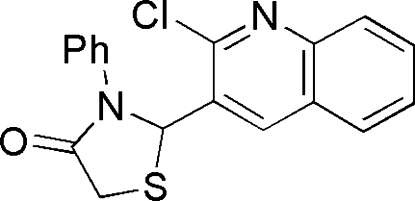


## Experimental

### 

#### Crystal data

C_18_H_13_ClN_2_OS
*M* *_r_* = 340.81
Monoclinic, 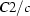

*a* = 16.1192 (6) Å
*b* = 12.7502 (5) Å
*c* = 16.8949 (6) Å
β = 110.379 (2)°
*V* = 3255.0 (2) Å^3^
*Z* = 8
Mo *K*α radiation
μ = 0.37 mm^−1^
*T* = 296 K
0.35 × 0.20 × 0.15 mm

#### Data collection

Bruker SMART CCD area-detector diffractometer
Absorption correction: none
12810 measured reflections
2883 independent reflections
2165 reflections with *I* > 2σ(*I*)
*R*_int_ = 0.027

#### Refinement

*R*[*F* ^2^ > 2σ(*F* ^2^)] = 0.037
*wR*(*F* ^2^) = 0.098
*S* = 1.05
2883 reflections
208 parameters
H-atom parameters constrained
Δρ_max_ = 0.22 e Å^−3^
Δρ_min_ = −0.24 e Å^−3^

### 

Data collection: *SMART* (Bruker, 2001 ▶); cell refinement: *SAINT* (Bruker, 2001 ▶); data reduction: *SAINT*; program(s) used to solve structure: *SHELXS97* (Sheldrick, 2008 ▶); program(s) used to refine structure: *SHELXL97* (Sheldrick, 2008 ▶); molecular graphics: *SHELXTL* (Sheldrick, 2008 ▶); software used to prepare material for publication: *SHELXTL*.

## Supplementary Material

Crystal structure: contains datablocks global, I. DOI: 10.1107/S1600536809041543/gw2069sup1.cif

Structure factors: contains datablocks I. DOI: 10.1107/S1600536809041543/gw2069Isup2.hkl

Additional supplementary materials:  crystallographic information; 3D view; checkCIF report

## Figures and Tables

**Table 1:** Hydrogen-bond geometry (Å, °)

*D*—H⋯*A*	*D*—H	H⋯*A*	*D*⋯*A*	*D*—H⋯*A*
C8—H8*A*⋯N1^i^	0.93	2.63	3.514 (3)	158
C3—H3*A*⋯O1^ii^	0.93	2.35	3.192 (2)	151

Symmetry codes: (i) 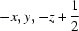; (ii) 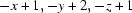.

**Fig. 1.**
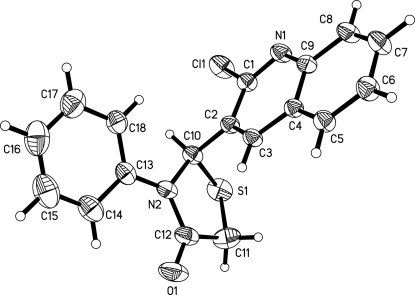
The molecular structure drawing for (I) showing 50% probability of displacement ellipsoids and the atom-numbering scheme.

**Fig. 2.**
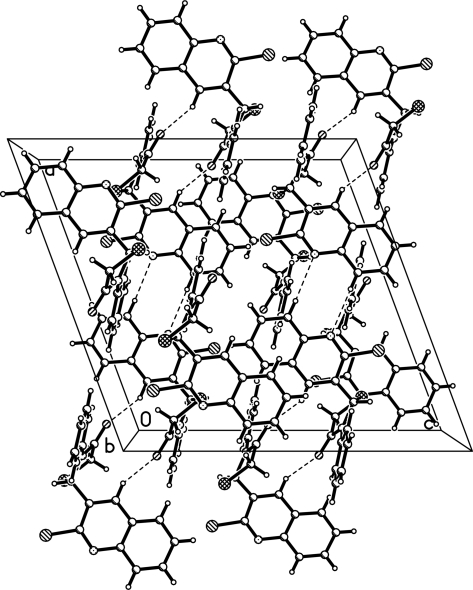
The molecular packing diagram of (I). The broken lines indicate hydrogen bonds.

## supplementary crystallographic information

### Comment

Thiazolidinone derivatives are important heterocyclic nitrogen compounds which display a wide range of biological activity. Some synthetic thiazolidinones have been used as antiviral (Abd Elhafez *et al.*, 2003), antioxidant (Shih and Ke, 2004), antimycobacterial (Kuecuekguezel *et al.*, 2006), antimicrobial (Subudhi *et al.*, 2007), and also as antiinflammatory (Srivastava *et al.*, 2006). We report here the structure of 2-(2-chloroquinolin-3-yl)- 3-phenylthiazolidin-4-one, (I).

In (I), the thiazolidinone ring is slightly distorted and adopts a envelope conformation: the atoms of C11, C12, N2 and C10 are coplanar, with S1 deviating from the defined plane by 0.673 Å. The basal plane is nearly perpendicular to the quinoline ring, forming a dihedral angle of 86.1 (1) °, and makes a dihedral angle of 14.9 (1) ° to benzene ring. The benzene ring is also nearly to perpendicular to the quinoline ring, forming a dihedral angle of 89.4 (1) °.

There are two non-classical hydrogen bonds of C3—H3A···O1 and C8—H8A···N1 in the crystal structure. The former links the adjacent molecules forming dimmers, while the latter also links another adjacent molecules forming polymers. The two above mentioned non-classical hydrogen bonds link the molecules forming polymers along b.

### Experimental

A solution of 2-chloroquinoline-3-carbadehyde (1.92 g, 10 mmol) and 5 mmol aniline (0.5 ml, 5.5 mmol) in anhydrous THF (30 ml) was stirred under ice-cold conditions for 5 min, followed by addition of mercapto acid (1.1 ml, 15 mmol). Dicyclohexylcarbodiimide (DCC) (6 mmol) was added to the reaction mixture 5 min later, the resulting mixture was stirred at ambient temperature for 1 h. Dicyclohexylurea (DCU) was removed by filtration and the filtrate was concentrated under reduced pressure and the residue was taken up in some ethyl acetate. The organic layer was successively washed with 5% aq. citric acid, water, 5% aq. sodium hydrogen carbonate, and then finally with brine. The organic layer was dried over magnesium sulfate and the solvent was removed under reduced pressure to get a crude product that was purified by column chromatography on silica gel with petroleum ether and ethyl acetate as eluents for stepwise elution. The colorless single crystals of the title compound suitable for X-raycrystallographic analysis were obtained by recrystallization from a mixture of petroleum ether and ethyl acetate. m.p.426–428 K.

### Refinement

The H atoms were calculated geometrically and refined as riding, with C—H = 0.93–0.98 Å. with *U*_iso_((C_methyl_)) = 1.5*U*_eq_; *U*_iso_(H) = 1.2*U*_eq_(parent atom).

### Crystal data

**Table:**

C_18_H_13_ClN_2_OS	*F*(000) = 1408
*M**_r_* = 340.81	*D*_x_ = 1.391 Mg m^−^^3^
Monoclinic, *C*2/*c*	Melting point = 426–428 K
Hall symbol: -C 2yc	Mo *K*α radiation, λ = 0.71073 Å
*a* = 16.1192 (6) Å	Cell parameters from 3808 reflections
*b* = 12.7502 (5) Å	θ = 2.7–26.3°
*c* = 16.8949 (6) Å	µ = 0.37 mm^−^^1^
β = 110.379 (2)°	*T* = 296 K
*V* = 3255.0 (2) Å^3^	Block, pale yellow
*Z* = 8	0.35 × 0.20 × 0.15 mm

### Data collection

**Table:**

Bruker SMART CCD area-detector diffractometer	2165 reflections with *I* > 2σ(*I*)
Radiation source: fine-focus sealed tube	*R*_int_ = 0.027
graphite	θ_max_ = 25.0°, θ_min_ = 2.1°
φ and ω scans	*h* = −19→19
12810 measured reflections	*k* = −14→15
2883 independent reflections	*l* = −20→20

### Refinement

**Table:**

Refinement on *F*^2^	Primary atom site location: structure-invariant direct methods
Least-squares matrix: full	Secondary atom site location: difference Fourier map
*R*[*F*^2^ > 2σ(*F*^2^)] = 0.037	Hydrogen site location: inferred from neighbouring sites
*wR*(*F*^2^) = 0.098	H-atom parameters constrained
*S* = 1.05	*w* = 1/[σ^2^(*F*_o_^2^) + (0.0404*P*)^2^ + 1.9966*P*] where *P* = (*F*_o_^2^ + 2*F*_c_^2^)/3
2883 reflections	(Δ/σ)_max_ = 0.001
208 parameters	Δρ_max_ = 0.22 e Å^−^^3^
0 restraints	Δρ_min_ = −0.24 e Å^−^^3^

### Special details

**Table:**

Geometry. All e.s.d.'s (except the e.s.d. in the dihedral angle between two l.s. planes) are estimated using the full covariance matrix. The cell e.s.d.'s are taken into account individually in the estimation of e.s.d.'s in distances, angles and torsion angles; correlations between e.s.d.'s in cell parameters are only used when they are defined by crystal symmetry. An approximate (isotropic) treatment of cell e.s.d.'s is used for estimating e.s.d.'s involving l.s. planes.
Refinement. Refinement of *F*^2^ against ALL reflections. The weighted *R*-factor *wR* and goodness of fit *S* are based on *F*^2^, conventional *R*-factors *R* are based on *F*, with *F* set to zero for negative *F*^2^. The threshold expression of *F*^2^ > σ(*F*^2^) is used only for calculating *R*-factors(gt) *etc*. and is not relevant to the choice of reflections for refinement. *R*-factors based on *F*^2^ are statistically about twice as large as those based on *F*, and *R*- factors based on ALL data will be even larger.

### Fractional atomic coordinates and isotropic or equivalent isotropic displacement parameters (Å^2^)

**Table:**

	*x*	*y*	*z*	*U*_iso_*/*U*_eq_	
Cl1	0.18759 (4)	0.86407 (6)	0.14286 (3)	0.0776 (2)	
S1	0.35490 (4)	1.06043 (5)	0.23906 (4)	0.0743 (2)	
N1	0.13806 (10)	0.84780 (14)	0.27204 (10)	0.0529 (4)	
C5	0.23728 (14)	0.88458 (16)	0.50321 (12)	0.0505 (5)	
H5A	0.2911	0.9013	0.5446	0.061*	
C2	0.29190 (12)	0.89607 (15)	0.30682 (11)	0.0432 (5)	
C1	0.20616 (13)	0.86948 (16)	0.25139 (11)	0.0486 (5)	
N2	0.45307 (10)	0.91604 (14)	0.33821 (9)	0.0483 (4)	
C9	0.14837 (12)	0.85342 (15)	0.35618 (12)	0.0455 (5)	
C3	0.30220 (12)	0.89873 (15)	0.39034 (11)	0.0433 (5)	
H3A	0.3576	0.9137	0.4300	0.052*	
C4	0.23063 (12)	0.87924 (15)	0.41773 (11)	0.0418 (4)	
C8	0.07547 (14)	0.83422 (19)	0.38073 (13)	0.0581 (6)	
H8A	0.0210	0.8175	0.3403	0.070*	
C10	0.36487 (12)	0.92437 (17)	0.27408 (11)	0.0488 (5)	
H10A	0.3613	0.8785	0.2265	0.059*	
C13	0.49430 (13)	0.81620 (18)	0.35847 (12)	0.0516 (5)	
C6	0.16534 (14)	0.86545 (18)	0.52517 (13)	0.0578 (6)	
H6A	0.1701	0.8693	0.5816	0.069*	
C7	0.08402 (14)	0.8399 (2)	0.46357 (14)	0.0635 (6)	
H7A	0.0353	0.8268	0.4795	0.076*	
O1	0.55375 (11)	1.01688 (16)	0.43920 (10)	0.0849 (6)	
C18	0.44621 (16)	0.72616 (19)	0.32907 (14)	0.0624 (6)	
H18A	0.3863	0.7308	0.2969	0.075*	
C12	0.48571 (15)	1.0079 (2)	0.37932 (14)	0.0613 (6)	
C14	0.58436 (16)	0.8072 (2)	0.40572 (14)	0.0731 (7)	
H14A	0.6184	0.8669	0.4258	0.088*	
C11	0.42542 (19)	1.0985 (2)	0.34260 (17)	0.0841 (8)	
H11A	0.4596	1.1600	0.3396	0.101*	
H11B	0.3904	1.1150	0.3775	0.101*	
C16	0.5747 (2)	0.6209 (3)	0.3948 (2)	0.0937 (9)	
H16A	0.6015	0.5556	0.4082	0.112*	
C17	0.4867 (2)	0.6282 (2)	0.34713 (18)	0.0835 (8)	
H17A	0.4539	0.5677	0.3268	0.100*	
C15	0.6223 (2)	0.7092 (3)	0.42233 (18)	0.0950 (10)	
H15A	0.6824	0.7036	0.4534	0.114*	

### Atomic displacement parameters (Å^2^)

**Table:**

	*U*^11^	*U*^22^	*U*^33^	*U*^12^	*U*^13^	*U*^23^
Cl1	0.0609 (4)	0.1297 (6)	0.0347 (3)	−0.0162 (4)	0.0073 (2)	−0.0082 (3)
S1	0.0791 (5)	0.0764 (4)	0.0645 (4)	−0.0054 (4)	0.0214 (3)	0.0224 (3)
N1	0.0406 (9)	0.0694 (12)	0.0419 (9)	−0.0089 (9)	0.0060 (7)	−0.0041 (8)
C5	0.0442 (11)	0.0637 (13)	0.0414 (10)	−0.0034 (10)	0.0122 (9)	−0.0016 (10)
C2	0.0385 (10)	0.0500 (11)	0.0375 (9)	−0.0028 (9)	0.0086 (8)	−0.0009 (8)
C1	0.0451 (11)	0.0600 (13)	0.0352 (10)	−0.0032 (10)	0.0072 (9)	−0.0028 (9)
N2	0.0397 (9)	0.0629 (11)	0.0414 (8)	−0.0110 (8)	0.0131 (7)	−0.0021 (8)
C9	0.0404 (11)	0.0488 (12)	0.0443 (10)	−0.0034 (9)	0.0108 (9)	−0.0007 (9)
C3	0.0337 (10)	0.0538 (12)	0.0380 (10)	−0.0036 (9)	0.0069 (8)	−0.0020 (8)
C4	0.0379 (10)	0.0457 (11)	0.0392 (9)	−0.0010 (9)	0.0099 (8)	−0.0007 (8)
C8	0.0407 (11)	0.0739 (15)	0.0563 (13)	−0.0125 (11)	0.0128 (10)	−0.0010 (11)
C10	0.0444 (11)	0.0641 (13)	0.0360 (10)	−0.0066 (10)	0.0116 (8)	−0.0013 (9)
C13	0.0488 (12)	0.0720 (15)	0.0406 (10)	−0.0003 (11)	0.0237 (9)	0.0066 (10)
C6	0.0576 (13)	0.0723 (15)	0.0474 (11)	−0.0042 (12)	0.0233 (10)	0.0014 (10)
C7	0.0489 (13)	0.0824 (17)	0.0647 (14)	−0.0100 (12)	0.0266 (11)	0.0031 (12)
O1	0.0623 (10)	0.1135 (15)	0.0676 (10)	−0.0327 (10)	0.0083 (9)	−0.0206 (10)
C18	0.0603 (14)	0.0692 (16)	0.0676 (14)	−0.0016 (13)	0.0346 (12)	−0.0020 (12)
C12	0.0547 (13)	0.0780 (17)	0.0527 (12)	−0.0226 (13)	0.0204 (11)	−0.0058 (12)
C14	0.0569 (14)	0.099 (2)	0.0590 (14)	0.0021 (14)	0.0147 (11)	0.0118 (13)
C11	0.097 (2)	0.0620 (16)	0.0873 (18)	−0.0189 (15)	0.0247 (16)	−0.0036 (14)
C16	0.102 (3)	0.094 (2)	0.096 (2)	0.032 (2)	0.048 (2)	0.0265 (18)
C17	0.104 (2)	0.0750 (19)	0.090 (2)	0.0042 (17)	0.0571 (18)	0.0035 (15)
C15	0.0746 (19)	0.123 (3)	0.0808 (19)	0.029 (2)	0.0185 (15)	0.0279 (19)

### Geometric parameters (Å, °)

**Table:**

Cl1—C1	1.7534 (19)	C10—H10A	0.9800
S1—C11	1.790 (3)	C13—C18	1.377 (3)
S1—C10	1.822 (2)	C13—C14	1.397 (3)
N1—C1	1.292 (3)	C6—C7	1.399 (3)
N1—C9	1.374 (2)	C6—H6A	0.9300
C5—C6	1.357 (3)	C7—H7A	0.9300
C5—C4	1.412 (3)	O1—C12	1.211 (3)
C5—H5A	0.9300	C18—C17	1.393 (4)
C2—C3	1.363 (2)	C18—H18A	0.9300
C2—C1	1.414 (3)	C12—C11	1.497 (4)
C2—C10	1.508 (3)	C14—C15	1.377 (4)
N2—C12	1.370 (3)	C14—H14A	0.9300
N2—C13	1.422 (3)	C11—H11A	0.9700
N2—C10	1.461 (2)	C11—H11B	0.9700
C9—C8	1.397 (3)	C16—C15	1.349 (4)
C9—C4	1.410 (2)	C16—C17	1.369 (4)
C3—C4	1.407 (3)	C16—H16A	0.9300
C3—H3A	0.9300	C17—H17A	0.9300
C8—C7	1.360 (3)	C15—H15A	0.9300
C8—H8A	0.9300		
			
C11—S1—C10	89.23 (11)	C18—C13—N2	120.15 (19)
C1—N1—C9	117.38 (16)	C14—C13—N2	121.1 (2)
C6—C5—C4	120.21 (19)	C5—C6—C7	120.5 (2)
C6—C5—H5A	119.9	C5—C6—H6A	119.8
C4—C5—H5A	119.9	C7—C6—H6A	119.8
C3—C2—C1	115.48 (18)	C8—C7—C6	120.7 (2)
C3—C2—C10	123.01 (16)	C8—C7—H7A	119.6
C1—C2—C10	121.45 (16)	C6—C7—H7A	119.6
N1—C1—C2	126.77 (18)	C13—C18—C17	120.4 (2)
N1—C1—Cl1	114.97 (14)	C13—C18—H18A	119.8
C2—C1—Cl1	118.26 (16)	C17—C18—H18A	119.8
C12—N2—C13	125.37 (18)	O1—C12—N2	125.5 (2)
C12—N2—C10	114.62 (18)	O1—C12—C11	122.8 (2)
C13—N2—C10	119.76 (17)	N2—C12—C11	111.71 (19)
N1—C9—C8	119.12 (17)	C15—C14—C13	119.3 (3)
N1—C9—C4	121.27 (17)	C15—C14—H14A	120.3
C8—C9—C4	119.61 (18)	C13—C14—H14A	120.3
C2—C3—C4	121.21 (17)	C12—C11—S1	107.20 (18)
C2—C3—H3A	119.4	C12—C11—H11A	110.3
C4—C3—H3A	119.4	S1—C11—H11A	110.3
C3—C4—C9	117.83 (16)	C12—C11—H11B	110.3
C3—C4—C5	123.31 (17)	S1—C11—H11B	110.3
C9—C4—C5	118.86 (18)	H11A—C11—H11B	108.5
C7—C8—C9	120.12 (19)	C15—C16—C17	119.6 (3)
C7—C8—H8A	119.9	C15—C16—H16A	120.2
C9—C8—H8A	119.9	C17—C16—H16A	120.2
N2—C10—C2	113.12 (15)	C16—C17—C18	120.0 (3)
N2—C10—S1	105.26 (13)	C16—C17—H17A	120.0
C2—C10—S1	110.80 (14)	C18—C17—H17A	120.0
N2—C10—H10A	109.2	C16—C15—C14	121.9 (3)
C2—C10—H10A	109.2	C16—C15—H15A	119.1
S1—C10—H10A	109.2	C14—C15—H15A	119.1
C18—C13—C14	118.7 (2)		
			
C9—N1—C1—C2	1.8 (3)	C3—C2—C10—S1	−96.2 (2)
C9—N1—C1—Cl1	−178.42 (15)	C1—C2—C10—S1	80.8 (2)
C3—C2—C1—N1	−0.1 (3)	C11—S1—C10—N2	−31.25 (16)
C10—C2—C1—N1	−177.3 (2)	C11—S1—C10—C2	91.36 (16)
C3—C2—C1—Cl1	−179.85 (15)	C12—N2—C13—C18	162.71 (19)
C10—C2—C1—Cl1	2.9 (3)	C10—N2—C13—C18	−11.2 (3)
C1—N1—C9—C8	177.7 (2)	C12—N2—C13—C14	−19.5 (3)
C1—N1—C9—C4	−1.6 (3)	C10—N2—C13—C14	166.56 (19)
C1—C2—C3—C4	−1.9 (3)	C4—C5—C6—C7	0.2 (3)
C10—C2—C3—C4	175.26 (18)	C9—C8—C7—C6	0.3 (4)
C2—C3—C4—C9	2.0 (3)	C5—C6—C7—C8	−0.2 (4)
C2—C3—C4—C5	−177.78 (19)	C14—C13—C18—C17	0.8 (3)
N1—C9—C4—C3	−0.2 (3)	N2—C13—C18—C17	178.56 (19)
C8—C9—C4—C3	−179.55 (19)	C13—N2—C12—O1	−0.8 (3)
N1—C9—C4—C5	179.58 (19)	C10—N2—C12—O1	173.4 (2)
C8—C9—C4—C5	0.3 (3)	C13—N2—C12—C11	−179.38 (19)
C6—C5—C4—C3	179.6 (2)	C10—N2—C12—C11	−5.2 (3)
C6—C5—C4—C9	−0.2 (3)	C18—C13—C14—C15	−0.7 (3)
N1—C9—C8—C7	−179.6 (2)	N2—C13—C14—C15	−178.5 (2)
C4—C9—C8—C7	−0.3 (3)	O1—C12—C11—S1	162.11 (19)
C12—N2—C10—C2	−94.5 (2)	N2—C12—C11—S1	−19.3 (2)
C13—N2—C10—C2	80.0 (2)	C10—S1—C11—C12	28.95 (19)
C12—N2—C10—S1	26.58 (19)	C15—C16—C17—C18	−1.7 (4)
C13—N2—C10—S1	−158.88 (14)	C13—C18—C17—C16	0.4 (4)
C3—C2—C10—N2	21.7 (3)	C17—C16—C15—C14	1.7 (5)
C1—C2—C10—N2	−161.27 (18)	C13—C14—C15—C16	−0.5 (4)

### Hydrogen-bond geometry (Å, °)

**Table:**

*D*—H···*A*	*D*—H	H···*A*	*D*···*A*	*D*—H···*A*
C8—H8A···N1^i^	0.93	2.63	3.514 (3)	158
C3—H3A···O1^ii^	0.93	2.35	3.192 (2)	151

Symmetry codes: (i) −*x*, *y*, −*z*+1/2; (ii) −*x*+1, −*y*+2, −*z*+1.
